# Epigenetic Regulation of Interleukin 6 by Histone Acetylation in Macrophages and Its Role in Paraquat-Induced Pulmonary Fibrosis

**DOI:** 10.3389/fimmu.2016.00696

**Published:** 2017-01-30

**Authors:** Lingli Hu, Yanfang Yu, Huijie Huang, Hanting Fan, Li Hu, Caiyong Yin, Kai Li, David J. R. Fulton, Feng Chen

**Affiliations:** ^1^Department of Forensic Medicine, Nanjing Medical University, Nanjing, China; ^2^Vascular Biology Center, Augusta University, Augusta, GA, USA; ^3^Department of Pharmacology, Augusta University, Augusta, GA, USA

**Keywords:** paraquat, *IL-6*, epigenetics, histone acetylation, pulmonary fibrosis, forensic toxicology

## Abstract

Overexpression of interleukin 6 (*IL-6*) has been proposed to contribute to pulmonary fibrosis and other fibrotic diseases. However, the regulatory mechanisms and the role of *IL-6* in fibrosis remain poorly understood. Epigenetics refers to alterations of gene expression without changes in the DNA sequence. Alternation of chromatin accessibility by histone acetylation acts as a critical epigenetic mechanism to regulate various gene transcriptions. The goal of this study was to determine the impact of *IL-6* in paraquat (PQ)-induced pulmonary fibrosis and to explore whether the epigenetic regulations may play a role in transcriptional regulation of *IL-6*. In PQ-treated lungs and macrophages, we found that the mRNA and protein expression of *IL-6* was robustly increased in a time-dependent and a dose-dependent manner. Our data demonstrated that PQ-induced *IL-6* expression in macrophages plays a central role in pulmonary fibrosis through enhanced epithelial-to-mesenchymal transition (EMT). *IL-6* expression and its role to enhance PQ-induced pulmonary fibrosis were increased by histone deacetylase (HDAC) inhibition and prevented by histone acetyltransferase (HAT) inhibition. In addition, the ability of CRISPR-ON transcription activation system (CRISPR-ON) to promote transcription of *IL-6* was enhanced by HDAC inhibitor and blocked by HAT inhibitor. Chromatin immunoprecipitation experiments revealed that HDAC inhibitor increased histones activation marks H3K4me3 and H3K9ac at *IL-6* promoter regions. In conclusion, *IL-6* functioning through EMT in PQ-induced pulmonary fibrosis was regulated dynamically by HDAC and HAT both *in vitro* and *in vivo via* epigenetically regulating chromatin accessibility.

## Introduction

Pulmonary fibrosis is a chronic, progressive inflammatory disease of pulmonary interstitial, the occurrence of which is about 1/10,000 and the survival rate is 30–50% at 5 years after diagnosis (1–3). The main pathological change associated with this disease is lung interstitial, which is characterized by cell proliferation and extracellular matrix (ECM) excessive accumulation (2, 4). The mechanisms involved remain undefined; however, growing evidences indicate that inflammatory, epithelial injury, and apoptosis, especially epithelial-to-mesenchymal transition (EMT) may play an important role in the development of pulmonary fibrosis (5–8). EMT is a process that loss of epithelial cells characteristics (e.g., intercellular junctions and apical–basolateral polarity) but gain mesenchymal functions (e.g., produce ECM components, migration, and invasion). During the EMT process, the mesenchymal markers such as α-smooth muscle actin (α*-SMA*) and vimentin are overexpressed, while epithelial markers such as E-cadherin and cytokeratin are low expressed (7, 9–11). Paraquat (PQ, 1, 1′-dimethyl-4, 4′-bipyridinium) is a highly toxic herbicide, which is widely used in numerous developing countries around the world. Ingestion of PQ leads to multiple organ damage especially in the lung, including epithelial cell destruction, pulmonary edema, and inflammation, which are considered to be mediated by the overproduction of reactive oxygen species. PQ induced progressive pulmonary fibrosis, the most serious lung damage is often associated with high mortality, appears as early as several days to several weeks after PQ ingestion (12). However, the underlying molecular mechanisms of which remain elusive.

Interleukin 6 (*IL-6*), as a multifunctional cytokine, is involved in the pathogenesis of various autoimmune and chronic inflammatory diseases through different signaling pathways (13). It is produced by various cells, but the main sources are macrophages and monocytes (14, 15). Macrophage, a key component of immune responses, is a predominant regulator of inflammation in diseases. *IL-6* that is released from macrophages into the extracellular space can also influence fibrosis and inflammation *via* paracrine actions on other cell types. *IL-6* exerts its biological activities through IL-6R and gp130. When *IL-6* binds to mIL-6R (membrane-bound form of IL-6R), homodimerization of gp130 is induced and form a high-affinity functional receptor complex of *IL-6*, IL-6R and gp130. The homodimerization of receptor complex activates Janus kinases (JAKs) that then phosphorylate tyrosine residues in the cytoplasmic domain of gp130. The gp130-mediated JAK activation by *IL-6* triggers two main signaling pathways: the gp130 Tyr759-derived SHP-2/ERK MAPK pathway and the gap130 YXXQ-mediated JAK/STAT pathway (16). *IL-6* can simultaneously generate functionally distinct or sometimes contradictory signals through its receptor complex, IL-6Ralpha and gp130. The final physiological output is thought to be a consequence of the diverse signaling pathways generated by a given ligand (17). *IL-6* induction promotes collagen deposition in multiorgans such as kidney, heart, and skin (18). The cytokine *IL-6* is elevated in mice and humans with pulmonary fibrosis (17, 19–23). However, its impact on fibrosis and regulatory mechanisms are not well understood.

Epigenetics refer to alterations of gene expression without changes in the DNA sequence. It is an area of research that encompasses three main mechanisms: DNA methylation, histone modifications to the tails of histones, and also non-coding RNAs including long and short non-coding RNAs (24). These three mechanisms all seek to regulate gene expression. The new emphasis on epigenetics is related to the increasing production of drugs capable of interfering with epigenetic mechanisms and able to trigger better clinical responses. The role of epigenetics in pulmonary fibrosis is a very novel area of investigation and while epigenetic modifiers have been shown to influence pulmonary fibrosis and chronic obstructive pulmonary disease (COPD) in experimental models and human diseases (25–29).

Histone modification, as an epigenetic mechanism, including acetylation, methylation, phosphorylation, deamination, β-*N*-acetylglucosamine, ADP ribosylation, ubiquitination, and sumoylation of Histones, can change the charge of histones which subsequently affect the structure of chromatin to upregulate or downregulate gene expression. For example, histone acetylation, the most common alteration of histone, is catalyzed by histone acetyltransferases (HATs) and histone deacetylases (HDACs). While HATs transfer an acetyl group from acetyl CoA to the ε-amino group of lysine side chains, HDACs remove an acetyl group from the lysine tail (30). The acetylated state of histone neutralizes its positive charge which may weaken the interaction between histone and the DNA strands. Thereby acetylation results in the N-terminus of histones, due to its negatively charged phosphate backbone, to move away from the DNA strands (31). These changes lead to a more open chromatin structure thus upregulating gene expression. In general, increased acetylation of histone residues is thought to weaken the interaction with DNA and thus provide greater accessibility to regulatory elements of DNA, while deacetylation of histone provides transcriptional repression to regulatory elements of DNA. The *HDAC* enzymes are a multi-class with 18 members that are referred to as class I (*HDACs*1–3 and 8), class II (*HDACs* 4–7, 9–10), class III (*SIRT*1–7), and Class IV (*HDAC11*) on the basis of function and sequence homology (32). The inhibitions of *HDACs*, in theory, decrease histone deacetylation, provide greater accessibility to chromatin structure, and increase gene expression, while the inhibitions of HATs reduce gene expression. In contrast, the ability of *HDAC* inhibitors to modify gene expression is complex, previous studies demonstrated that approximately 30% of the transcriptome are regulated by *HDACs* with equal proportion of the upregulation and downregulation in gene expression, and the pattern and direction of changes in gene expression are different and depended on cell types (33, 34). This is partially due to the fact that *HDAC* inhibitors can significantly increase the deacetylation of histones at multiple genomic DNA regions (33). The mechanisms influencing *IL-6* expression in pulmonary fibrosis are not yet known, and epigenetic control of *IL-6* expression is, in general, poorly understood.

As a RNA-guided transcriptional activator system, CRISPR-ON is a novel and powerful tool that can effectively induce specific gene expression (35, 36). The CRISPR-ON consists of three components: a nucleolytically inactive Cas9–VP64 fusion, a single guide RNA (sgRNA) incorporating two MS2 RNA aptamers at the tetraloop and stem-loop, and the MS2-P65-HSF1 activation helper protein. With the ability to robustly activate coding and non-coding elements (lincRNA), CRISPR-ON could be used to regulate gene expression epigenetically (37).

Therefore, the aim of this study was to determine the impact of *IL-6* in PQ-induced pulmonary fibrosis and to explore whether the epigenetic regulators play a role in the transcriptional regulation of *IL-6*.

## Materials and Methods

### Animals

This study was carried out in accordance with the guidelines of Institute for Laboratory Animal Research of the Nanjing Medical University. The protocol was approved by the Animal Care and Ethical Committee of Nanjing Medical University. Male mice (C57BL/6 mice, Oriental Bio Service Inc., Nanjing), weighing 20–25 g (7–8 weeks) at beginning of experiment, were used. The animals were maintained in a constant environmental condition (temperature 23 ± 2°C, humidity 55 ± 5%, 12:12 h light/dark cycle) in the Animal Research Center of Nanjing Medical University. They had free access to food and water before and after all procedures.

### Treatment of Mice

Mice were treated with intraperitoneal (i.p.) injections of PQ (Sigma, St. Louis, MO, USA) 10 or 50 mg/kg, respectively (38, 39). Control mice were injected with the same volume of vehicle. Mice were harvested on day 3, and samples were used to study PQ-induced pulmonary acute inflammation.

Mice were treated with i.p. injections of low dose of PQ 10 mg/kg for 1 month. Control mice were injected with the same volume of vehicle. Mice were sacrificed and samples were collected at the end of this study to determine the effect of PQ on pulmonary fibrosis.

*In vivo* neutralization of *IL-6* trans-signaling was performed using recombinant gp130Fc chimera (R&D Systems, Minneapolis, MN, USA). Mice were injected with saline or PQ 10 mg/kg, respectively, for 33 days. Mice were treated with vehicle (200 µl sterile PBS) or gp130Fc (2 µg/mouse reconstituted in 200 µl sterile PBS) 1 h before and 18 days after PQ injection (18, 40), and then mice were sacrificed and samples were collected to assess changes in pulmonary phenotype.

To study HDAC and HAT inhibition effect on pulmonary acute inflammation, mice were treated with i.p. injections of PQ at 10 mg/kg for 3 days. Anacardic acid (Selleck Chemicals, Houston, TX, USA) is a potent inhibitor of HAT, and VPA is an antagonist of HDAC. Valproic acid sodium salt (VPA, Selleck Chemicals, Houston, TX, USA) (3.5 mg/kg) (41) or anacardic acid (5 mg/kg) (42, 43) was injected 24 h and 1 h before PQ injection. Control mice were injected with the same volume of vehicle. To study HAT inhibition effect on pulmonary fibrosis, mice were treated with i.p. injections of PQ at 10 mg/kg for 1 month. Anacardic acid (5 mg/kg) was given 1 h before PQ injection and 24 h and 15 days after PQ injection. Control mice were injected with the same volume of vehicle. Samples were collected on day 30 after PQ injection.

### Isolation of Bone Marrow-Derived Macrophages

The femurs of male C57BL/6 mice (7–8 weeks old) were isolated and removed after euthanasia. Bone marrow cavities were flushed with PBS with 5% penicillin–streptomycin. Flushed cells were pelleted and resuspended in RPMI 1640 media (Hyclone, South Logan, UT, USA) supplemented with 10% FBS and 30% L929 media supplement. Then, flushed cells were plated in six-well plates at a density of 1.5–2 × 10^6^ cells/well and incubated at 37°C, 5% CO_2_. On day 7, macrophages were treated for experiment.

To make L929 media supplement, L929 cells were plated at a density of 4 × 10^6^ cells/100-mm cell culture dishes in 10 ml media consisting of RPMI 1640 with 1% penicillin–streptomycin, 1% l-glutamine, and 10% FBS.

### Cell Culture and Treatment

To study the effect of PQ on macrophages, cells were incubated with vehicle or increasing concentration of PQ 20, 40, 60, and 80 µM for 24 h. For time course experiment, cells were treated with PQ 80 µM, and samples collected on 4, 8, 12, and 24 h. For HDAC and HAT inhibition experiment, macrophages were treated with VPA (1 mM) (44) or anacardic acid (25 µM) (45) 1 h ahead of PQ 80 µM treatment.

For *in vitro* activation of IL-6 trans-signaling experiment, 16HBE and 3T3 were treated with recombinant IL-6 10 ng/ml (R&D Systems, Minneapolis, MN, USA) for 48 h (46).

### Immunofluorescence

*Collagen1-*α immunofluorescence was performed in paraffin-embedded sections of mouse lung. Before immunostaining, antigen retrieval was performed by heating slides in pH 6.0 citrate buffer at 100°C for 20 min in a microwave oven at 500 W using antigen retrieval solution (10 mM Tris and 1 mM EDTA, pH 9.0). Non-specific antibody binding was blocked for 20 min by incubation with 0.05% w/v BSA in PBS. Slices were stained using the anti-collagen1-α antibody (Novus, Littleton, CO, USA, 1:100 dilution) at 4°C overnight followed by staining with donkey-anti-rabbit secondary antibody (Santa Cruz, CA, USA, 1:200 dilution) at 37°C for 2 h.

### Hematoxylin and Eosin (H&E) Staining and Masson’s Trichrome

Mouse lungs were harvested and placed in 4% paraformaldehyde for 48 h, then embedded in paraffin and cut into 5 μm-thick serial sections, finally stained with H&E or Masson’s trichrome.

### Analysis of mRNA Expression

Total RNA from whole-lung and cells were extracted using TRIZOL (Takara Bio, Kusatsu, Shiga, Japan) following the manufacturer’s instruction and then used to synthesize cDNA using the iScriptcDNA Synthesis Kit (Bio-Rad, Hercules, CA, USA). Relative gene expression was determined using real-time RT-PCR with the following primers: Homo*IL-6*: TTCGGTCCAGTTGCCTTCTC (forward), GAGGTGAGTGGCTGTCTGTG (reverse). Mus*IL-6*: CCAATTTCCAATGCTCTCCT (forward), ACCACAGTGAGGAATGTCCA (reverse). Homo*TGF-*β: CCTGCCTGTCTGCACTATTC (forward), TGCCCAAGGTGCTCAATAAA (reverse). Mus *TGF-*β: CTGCTGACCCCCACTGATAC (forward), GTGAGCGCTGAATCGAAAGC (reverse). Homo α*-SMA*: GATGGTGGGAATGGGACAAA (forward), GCCATGTTCTATCGGGTACTTC (reverse). Mus α*-SMA*: GTACCACCATGTACCCAGGC (forward), GCTGGAAGGTAGACAGCGAA (reverse). Homo *COL1*α: CAAGAGGAAGGCCAAGTCGAGG (forward), CGTTGTCGCAGACGCAGAT (reverse). Mus *COL1-*α: CTGACGCATGGCCAAGAAGA (forward), TACCTCGGGTTTCCACGTCT (reverse). Homo *GREM1*: GCTAAAGAGAAGACGACGAGAG (forward), AGGGAGGTCATATCCCTTACA (reverse). Mus *GREM1*: CACTCGTCCACAGCGAAGAA (forward), TTGTGCTGAGCCTTGTCAGG (reverse). Homo*FN1*: TTGCTCCTGCACATGCTTTG (forward), CATGAAGCACTCAATTGGGCA (reverse). Homo *STAT3*: GCGGTAAGACCCAGATCCAG (forward), GGTCTTCAGGTATGGGGCAG (reverse). Homo *GAPDH*: AGAAGGCTGGGGCTCATTTG (forward), AGGGGCCATCCACAGTCTTC (reverse). Mus *GAPDH*: AGGTCATCCCAGAGCTGAACG (forward), CACCCTGTTGCTGTAGCCGTAT (reverse). Mus *18s*: CTCAACACGGGAAACCTCAC (forward), CGCTCCACCAACTAAGAACG (reverse). Mus *HDAC1*: CTCACCGAATCCGCATGACT (forward), ATTGGCTTTGTGAGGACGGT (reverse). Mus *HDAC2*: TATCCCGCTCTGTGCCCTAC (forward), GAGGCTTCATGGGATGACCC (reverse). Mus *HDAC3*: GGTGGCTACACTGTCCGAAA (forward), GGAGTGTGAAATCTGGGGCA (reverse). Mus *HDAC4*: GATGGACATCCACAGCAAGTA (forward), CTGTCTCAGCTTCTTCCTTCTC (reverse). Mus *HDAC5*: TTGACATCACAGCAGCTCCG (forward), ATGCCATCTGCCGACTCGTT (reverse). Mus *HDAC7*: GGGCTCTTCCAGAACAGATTAG (forward), CCGAGGCCAAGTTAAGAATAGT (reverse). Mus *HDAC8*: CCACCGAATCCAGCAAATCC (forward), CACAAACCGCTTGCATCAAC (reverse). Mus *IL-1*β: TCAGGCAGGCAGTATCACTC (forward), TCATCTCGGAGCCTGTAGTG (reverse). Mus *IL-8* TTGGTGATGCTGGTCATCTT (forward), TTTAGATGCAGCCCAGACAG (reverse). Mus *COX2*: AAGACTTGCCAGGCTGAACT (forward), CTTCTGCAGTCCAGGTTCAA (reverse). Mus *MMP9*: CCAGCCGACTTTTGTGGTCT (forward), TGGCCTTTAGTGTCTGGCTG (reverse). Mus *TNF-*α: GACAGTGACCTGGACTGTGG (forward), TGAGACAGAGGCAACCTGAC (reverse). Mus *VCAM1*: GCCTCAACGGTACTTTGGAT (forward), GTGGGCTGTCTATCTGGGTT (reverse).

### Western Blot of Lung Lysates

Whole-lung and cell protein were extracted using RIPA Lysis Buffer (Beyotime, Shanghai, China) according to the manufacturer’s instructions and then solubilized in 2× sample buffer. Protein concentration was determined with BCA Protein Assay Kit (Beyotime, Shanghai, China). Proteins were separated by SDS-polyacrylamide gel electrophoresis and transferred to polyvinylidene difluoride membrane (Bio-Rad, Hercules, CA, USA). The membranes were incubated with 5% non-fat dried milk for 60 min and then incubated with anti-α*-SMA* antibody (Sigma, St. Louis, MO, USA, 1:5,000 dilution) and anti-beta-actin antibody (Cell Signaling, Houston, TX, USA) at 4°C overnight. Then, the membranes were incubated with an HRP-labeled secondary antibody (Santa Cruz Biotechnology, CA, USA, 1:5,000 dilution) at 37°C for 1 h and developed using the ECL detection Kit.

### ELISA Analysis of *IL-6* in Macrophage Lysates and Serum

The level of *IL-6* was quantified in protein lysates purified from mice macrophages or serum using a mouse *IL-6* ELISA Kit (ExCell Bio, Taicang, China). Lysate (50 µl) or serum samples (50 µl) were subjected to ELISA analysis according to the manufacturer’s protocol.

### Engineered CRISPR–Cas9 and DNA Constructs

The use and design of engineered Cas9 complex and efficient sgRNA to induce *IL-6* transcriptional activation followed the protocol of Dr. Zhang (37). The sgRNA primers were annealed and cloned into sgRNA (MS2)-plasmids *via Bbs*I sites. All of the CRISPR constructs were purchased from Addgene (Cat: #61422, 61423, 61424, 61362, and 61358). The *IL-6* promoter-luciferase construct was generated by synthesizing the DNA fragment corresponding to *IL-6* promoter region (*IL-6* TSS −2,000 to 0) from GenScript and subcloning into pGL3-Basic vector.

### Chromatin Immunoprecipitation (ChIP)—Quantitative PCR (qPCR)

Chromatin immunoprecipitation was performed using the EZ ChIP™ Chromatin Immunoprecipitation Kit (Merck Millipore, Billerica, MA, USA) according to the manufacturer’s instructions. Macrophages were treated with vehicle (DMSO) or scriptaid (3 µg/ml) for 24 h and then fixed with 4% formaldehyde for 10 min. Chromatin was then prepared by enzymatic shearing and optimized according to the manufacturer’s instructions. ChIP was performed using ChIP-IT express on sheared chromatin from approximately 7.5 × 10^5^ cells using a negative control IgG, an anti-H3K4me3antibody, and an anti-H3K9ac antibody. Real-time PCR was performed on DNA isolated from each of the ChIP reactions using specific primer pairs for the mouse *IL-6* promoter regions (F: CACTTCACAAGTCGGAGGCT and R: AATGAATGGACGCCCACACT). The Δ*C*_t_ was calculated by the relative fold enrichment for each antibody used versus IgG negative control.

### Promoter Activity Assays

Cells were transfected and the total amount of expression plasmid transfected per well was balanced with varying amounts of a control vector. Firefly luciferase reporter plasmids and control luciferase plasmid (*Renilla* luciferase) were cotransfected into the cells, and 24 h post-transfection, cells were treated with PQ, VPA and anacardic acid for another 24 h. Cells were eventually processed in lysis buffer (Promega, Madison, WI, USA), and promoter activity was measured by a dual luciferase system using firefly luciferase normalized to *Renilla* luciferase (Promega, Madison, WI, USA).

### Statistical Analysis

Data are presented as mean ± SEM. Statistical analysis was performed using Instat software (GraphPad Software Inc., San Diego, CA, USA). An unpaired Student’s *t*-test and an ANOVA analysis with a Bonferroni *post hoc* test were used for single and multiple comparisons between two or more groups, respectively. The *P* < 0.05 was considered statistically significant.

## Results

### PQ-Induced Acute and Chronic Lung Injury and Increased *IL-6* Expression

**Figure 1:**
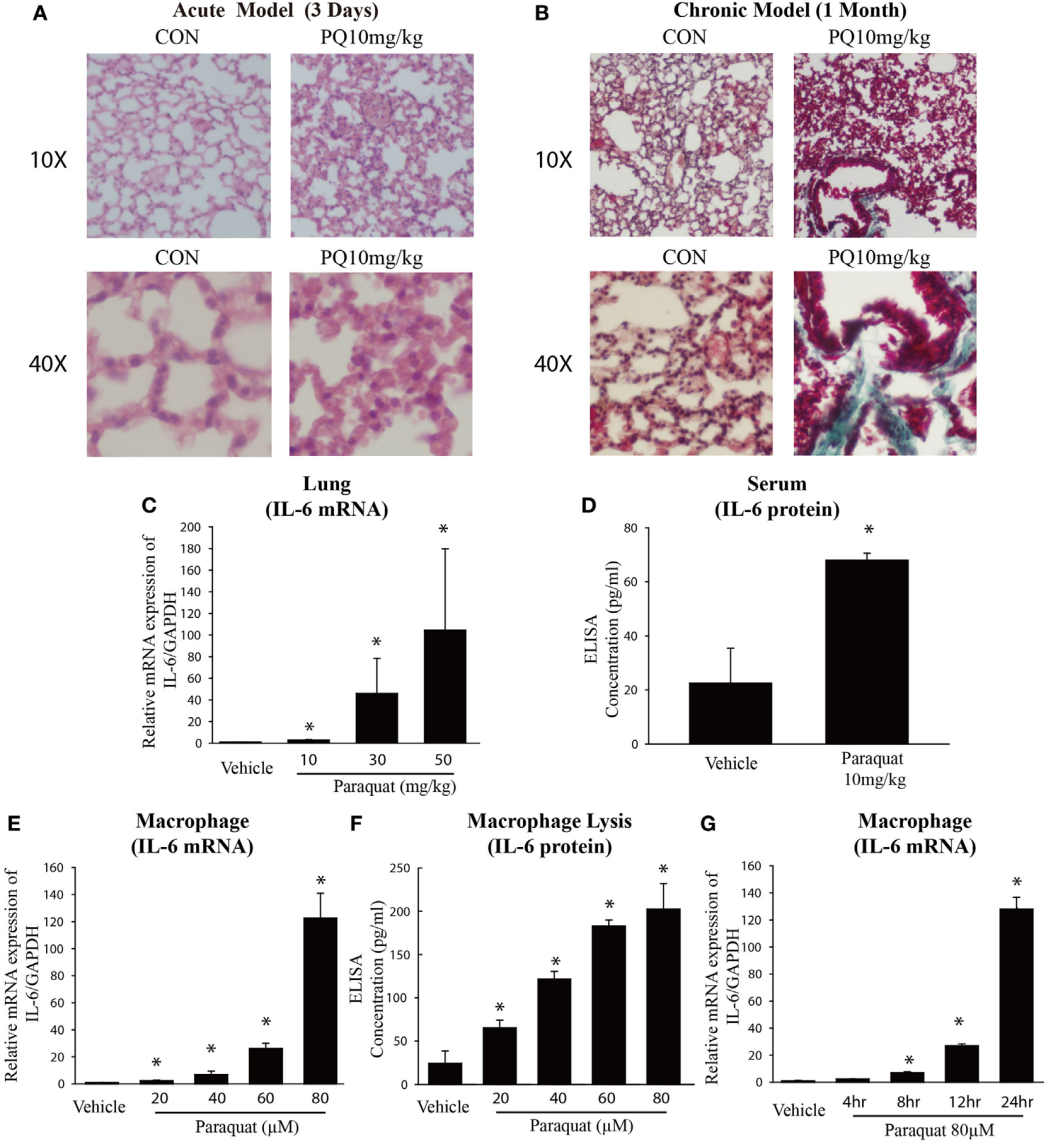
**Paraquat (PQ)-induced acute pulmonary inflammation, pulmonary fibrosis, and increased *IL-6* expression**. Wild-type C57BL/6 male mice were treated with vehicle (saline) or 10 mg/kg of PQ for 3 days or 1 month. **(A)** Lung sections from 3-day mice were stained with hematoxylin and eosin staining for visualization of inflammatory cell infiltration. **(B)** Lung sections from day 30 mice were stained with Masson’s trichrome for visualization of collagen deposition (green). **(C)** *IL-6* was measured in whole-lung RNA of 3-day mice. **(D)** ELISA measurement of *IL-6* in the serum of day 3 mice. **(E)** Macrophages were treated with increasing concentrations of PQ for 24 h, and *IL-6* mRNA expression was determined by real-time PCR. **(F)** ELISA measurement of *IL-6* in lysates of macrophages. **(G)** Macrophages were treated with PQ (80 µM) for 0, 4, 8, 12, and 24 h, and *IL-6* mRNA expression was determined by real-time PCR. Sections are representative of *n* ≥ 5 mice from each group. Macrophages were plated in six-well plates at a density of 1.5–2 × 10^6^ cells/well, and the data are representative of *n* > 5 from each group. Data are expressed as means ± SEM, *P < 0.05 versus vehicle.

We examined the effect of PQ-induced acute lung injury and chronic pulmonary fibrosis by performing H&E and Masson’s trichrome staining in the lung sections from PQ treated mice for 3 days and 1 month. As shown in Figure 1A, we found that inflammatory cells were infiltrated into lung structure in mice treated with PQ. With the prolongation of PQ treatment, the amount of collagen fibers was increased indicated by Masson’s trichrome staining (Figure 1B). As the exposure to PQ leads to significant inflammation and fibrosis in the lung, we next investigated the effect of PQ on the level of inflammation-related gene expression *in vivo* and *in vitro*. We found that PQ potently increases the mRNA and protein expression of *IL-6* in a dose-dependent manner in the lung and serum following PQ exposure (Figures 1C,D). While we did not observed a significant change in the level of *IL-1*β, *IL-8, COX-2, MMP9, TNF-*α, and *VCAM1* gene expression (Figures S1A–F in Supplementary Material). Since *IL-6* is mainly produced in immune cells and fibroblasts, we then measured *IL-6* mRNA and protein expression in macrophages and found that PQ significantly increased the mRNA and protein expression of *IL-6* in a dose-dependent and time-dependent manner in macrophages (Figures 1E–G). However, the level of *IL-6* remains unchanged in human lung fibroblast and epithelial cells 16HBE (Figures S2A,B in Supplementary Material).

### Involvement of EMT and Associated *IL-6* in PQ-Induced Pulmonary Fibrosis

**Figure 2:**
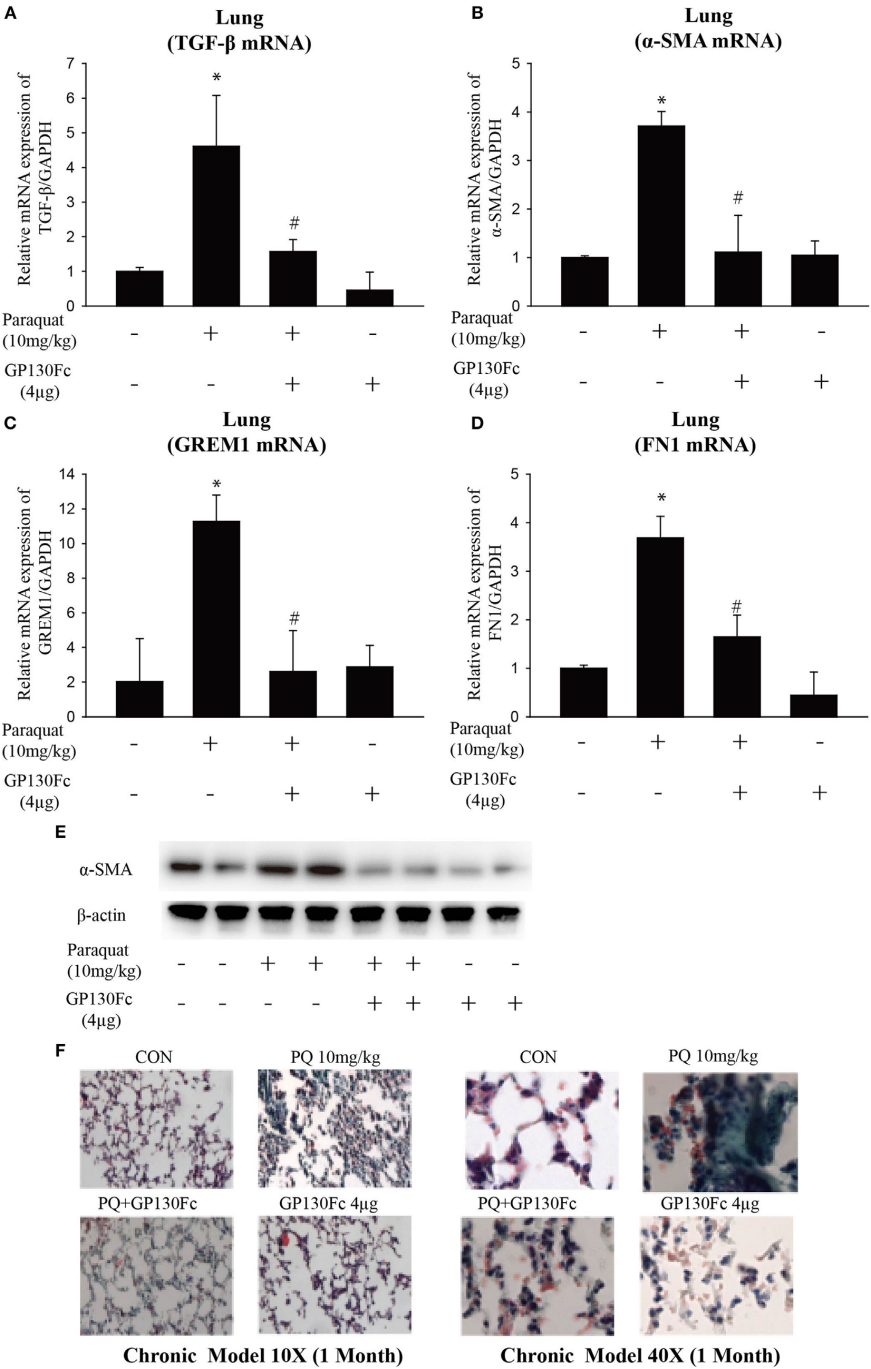
**GP130Fc downregulated the expression of fibrotic markers and ameliorated paraquat (PQ)-induced pulmonary fibrosis**. Wild-type mice were injected with saline or PQ 10 mg/kg, respectively, for 33 days. Mice were treated with vehicle (200 µl sterile PBS) or gp130Fc (2 µg/mouse reconstituted in 200 µl sterile PBS) 1 h before and 18 days after PQ injection. **(A–D)** The mRNA expression of *TGF-*β, α-smooth muscle actin (α*-SMA*), *GREM1*, and *FN1* were measured in whole-lung RNA. **(E)** Western blot analysis of α*-SMA* expression in whole-lung lysates. **(F)** Masson’s trichrome for collagen deposition (green). Representative images of *n* = 4–6 mice from each group are shown. Data are expressed as means ± SEM, *P < 0.05 versus vehicle and ^#^*P* < 0.05 versus PQ alone.

**Figure 3:**
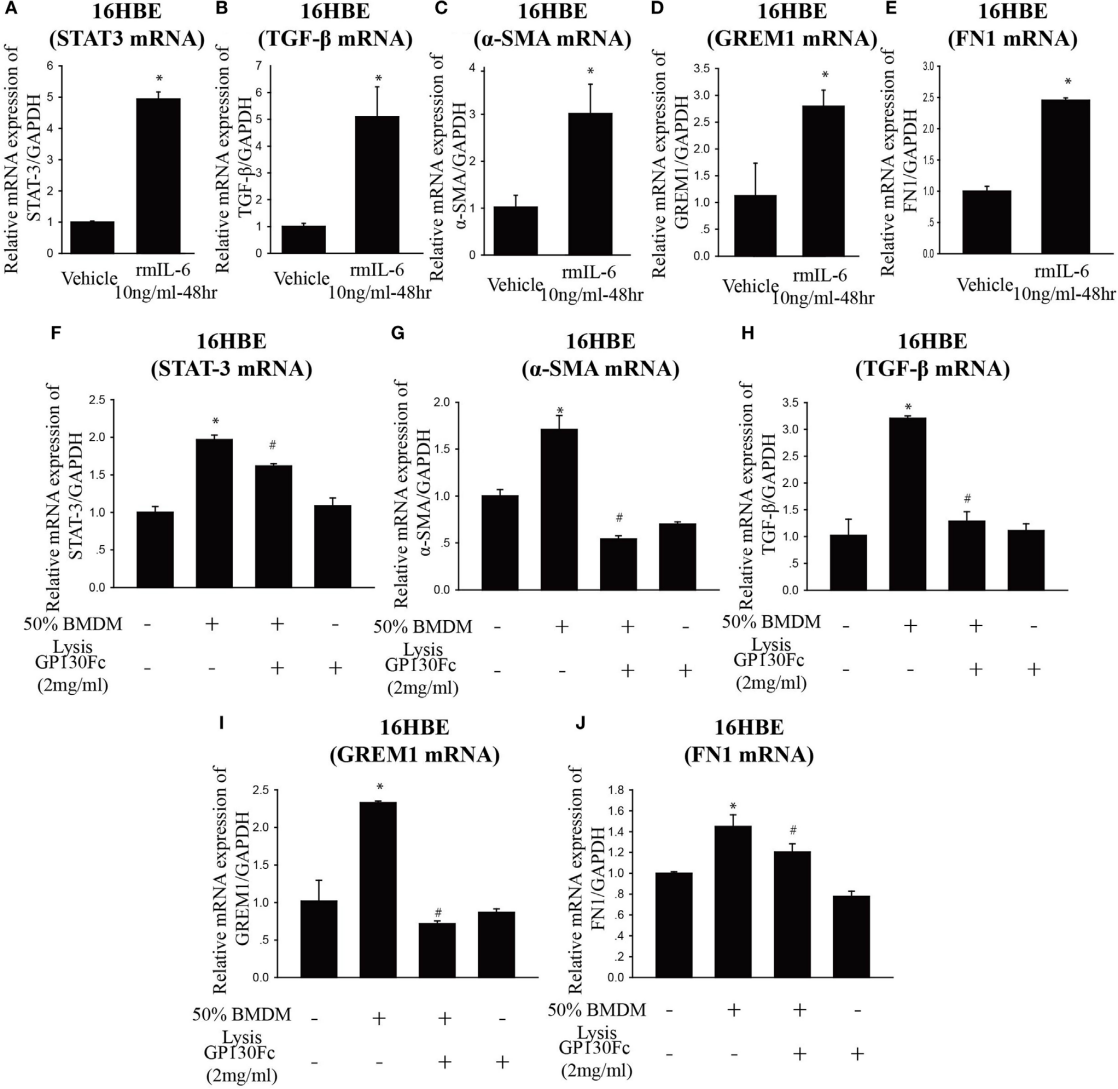
**Paraquat (PQ)-induced pulmonary fibrosis through epithelial-to-mesenchymal transition**. **(A–E)** 16HBE cells were treated with vehicle or recombinant IL-6 10 ng/ml for 48 h. *STAT-3, TGF-*β, α-smooth muscle actin (α*-SMA*), *GREM1*, and *FN1* mRNA expression were determined by real-time PCR. Bar graphs show the normalized levels of *STAT-3, TGF-*β, α*-SMA, GREM1*, and *FN1* by *GAPDH*. **(F–J)** 16HBE were incubated with or without 50% macrophage lysates that were treated with or without PQ or recombinant gp130Fc. S*TAT-3*, α*-SMA, TGF-*β, *GREM1*, and *FN1* mRNA expression were determined by real-time PCR. 16HBE cells were plated in six-well plates at a density of 1.5–2 × 10^6^ cells/well, and the data are representative of *n* > 5 from each group. *P < 0.05 versus vehicle and ^#^*P* < 0.05 versus 50% macrophage lysates (*n* = 4–6).

To determine the functional role of *IL-6* in PQ-induced pulmonary fibrosis, we next blocked *IL-6* trans-signaling with recombinant soluble gp130Fc. PQ exposure significantly increased the expression level of *TGF-*β, α*-SMA, GREM1*, and *FN1* in mouse lung, which were reduced in the presence of recombinant gp130Fc (Figures 2A–E). In addition, *in vivo* neutralization of *IL-6* trans-signaling by using recombinant gp130Fc attenuated pulmonary fibrosis phenotype (Figure 2F). *IL-6* that is released from macrophages into the extracellular space can also influence fibrosis and inflammation *via* paracrine actions on other cell types. We next treated mice fibroblasts with recombinant *IL-6* protein and the fibrotic gene expression including α*-SMA, GREM1*, and *FN1* were unchanged (Figures S3A–C in Supplementary Material). To explore the role of *IL-6* in the pathogenic mechanism of PQ-induced pulmonary fibrosis, we tested the levels of EMT-related genes in epithelial cells. And we found EMT-related genes such as *STAT3, TGF-*β, α*-SMA, GREM1*, and *FN1* were increased in association with the treatment of recombinant *IL-6* in 16HBE (Figures 3A–E). To further understand the role of *IL-6* in mediating macrophage and epithelial cell interaction, we treated 16HBE with PQ-treated macrophage lysates in the presence or absence of recombinant gp130Fc. These data showed that the expression of EMT-related genes were elevated in 16HBE following PQ-treated macrophage lysates but reduced in the presence of recombinant gp130Fc (Figures 3F–J). Together, our data demonstrated that PQ-induced *IL-6* expression in macrophages plays a central role in pulmonary fibrosis through enhanced EMT transition.

### *IL-6* Expression Is Associated with Histone Acetylation, and HAT Inhibition Mitigates PQ-Induced Pulmonary Fibrosis

**Figure 4:**
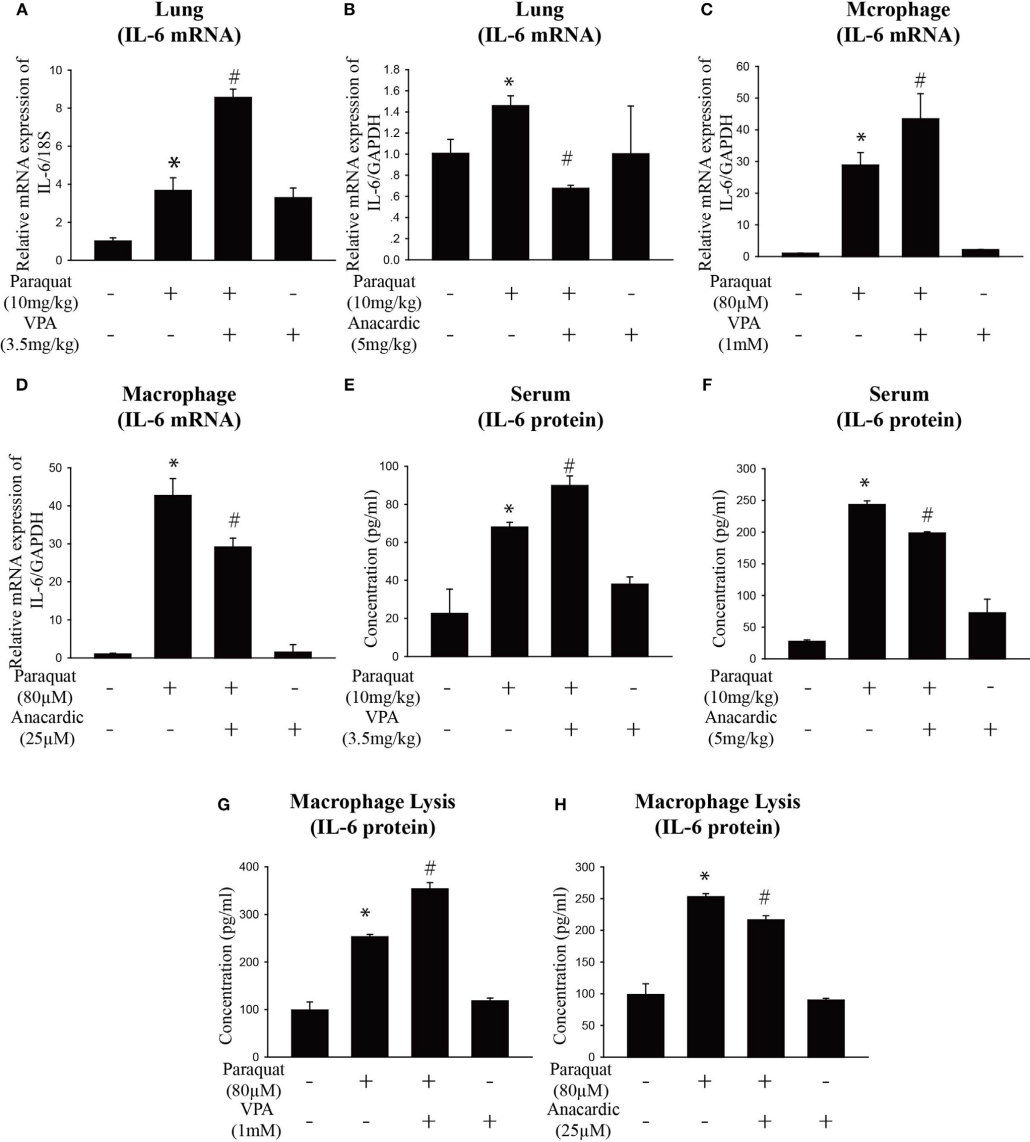
**Histone acetylation regulates *IL-6* expression**. **(A,B)** Wild-type C57BL/6 male mice were treated with vehicle (saline) or 10 mg/kg of paraquat (PQ) for 3 days, and mice received VPA (3.5 mg/kg) or anacardic acid (5 mg/kg) starting 24 and 1 h before PQ injection. *IL-6* mRNA expression was determined by real-time PCR. **(C,D)** Macrophages were treated with vehicle or PQ (80 µM) for 24 h, and cells received VPA (1 mM) or anacardic acid (25 µM) starting 1 h before PQ treatment. *IL-6* mRNA expression was determined by real-time PCR. **(E–H)** ELISA measurement of *IL-6* in serum of day 3 mice and in lysates of macrophages in the above mentioned experimental groups. Data are expressed as means ± SEM, *P < 0.05 versus vehicle and ^#^*P* < 0.05 versus PQ (*n* = 4–6).

**Figure 5:**
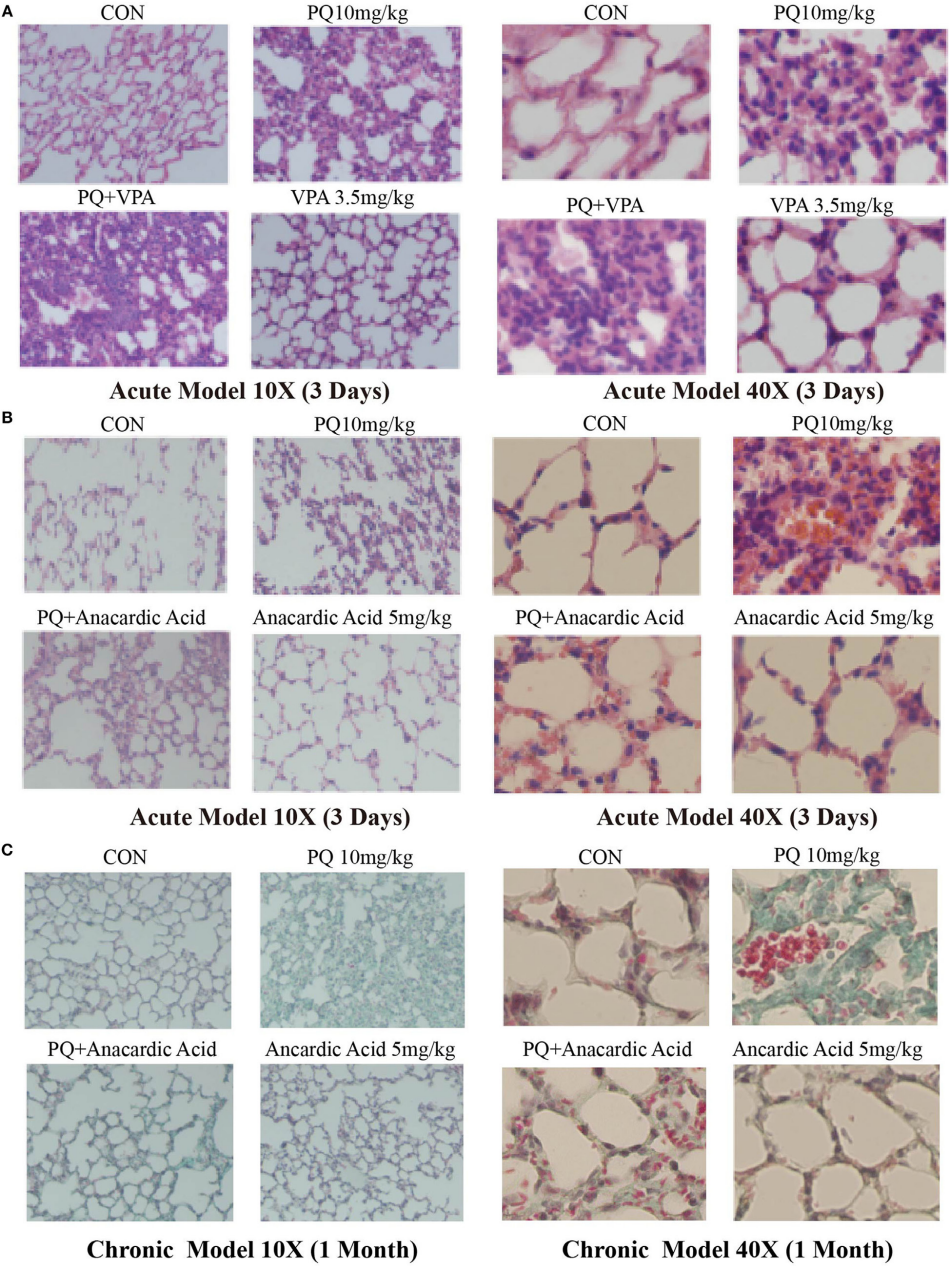

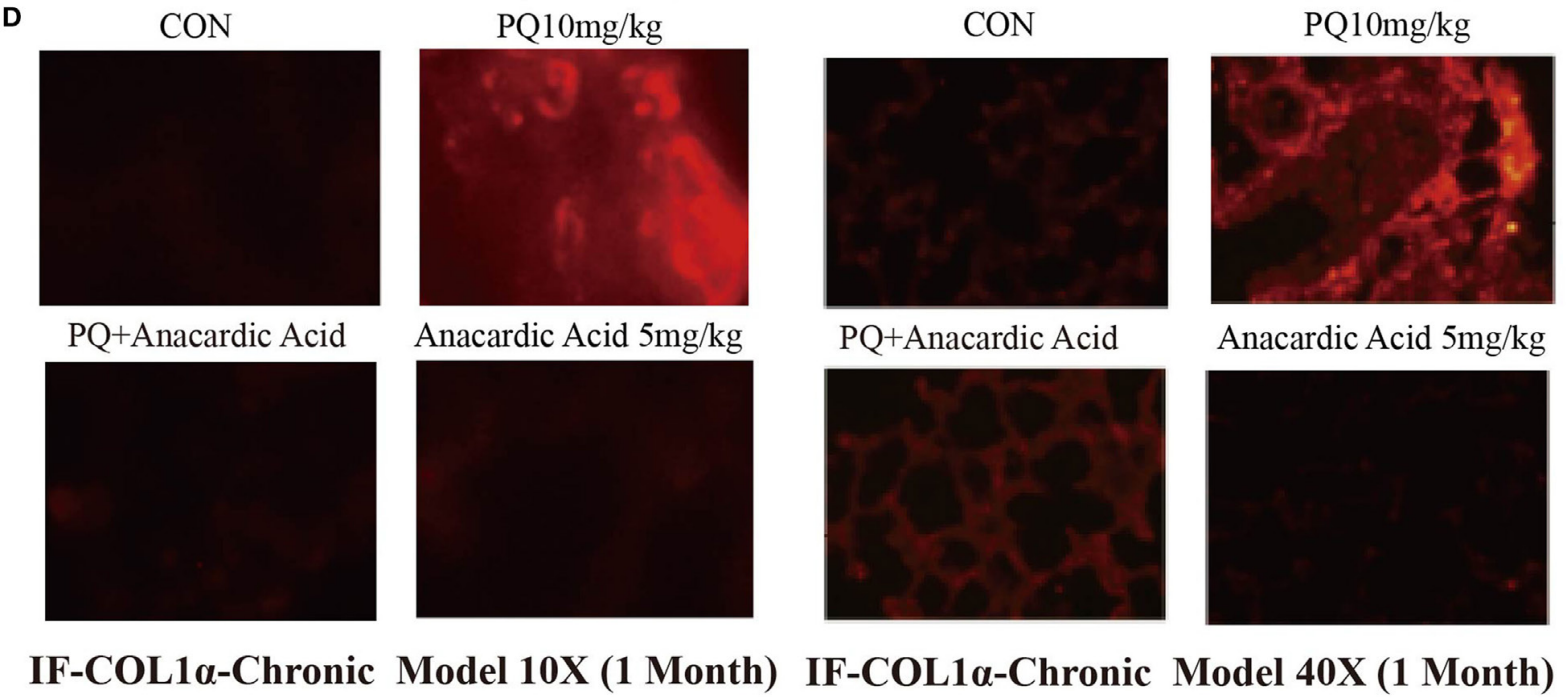
**Changes in pulmonary acute inflammation, pulmonary fibrosis following paraquat (PQ) exposure in mice treated with histone deacetylase or histone acetyltransferase inhibitors**. Wild-type C57BL/6 male mice were treated with vehicle (saline) or 10 mg/kg of PQ for 3 days, and mice received VPA (3.5 mg/kg) or anacardic acid (5 mg/kg) starting 24 and 1 h before PQ injection. **(A,B)** Lung sections from day 3 mice were stained with hematoxylin and eosin staining for visualization of inflammatory cell infiltration. Wild-type C57BL/6 male mice were treated with vehicle (saline) or 10 mg/kg of PQ for1 month, and mice received anacardic acid (5 mg/kg) starting 1 h before PQ injection, 24 h and 15 days after PQ injection. **(C)** Lung sections from day 30 mice were stained with Masson’s trichrome for visualization of collagen deposition (green). **(D)** *COL1-*α immunofluorescence (red) in lung sections. Sections are representative of *n* = 4–6 mice from each group.

**Figure 6:**
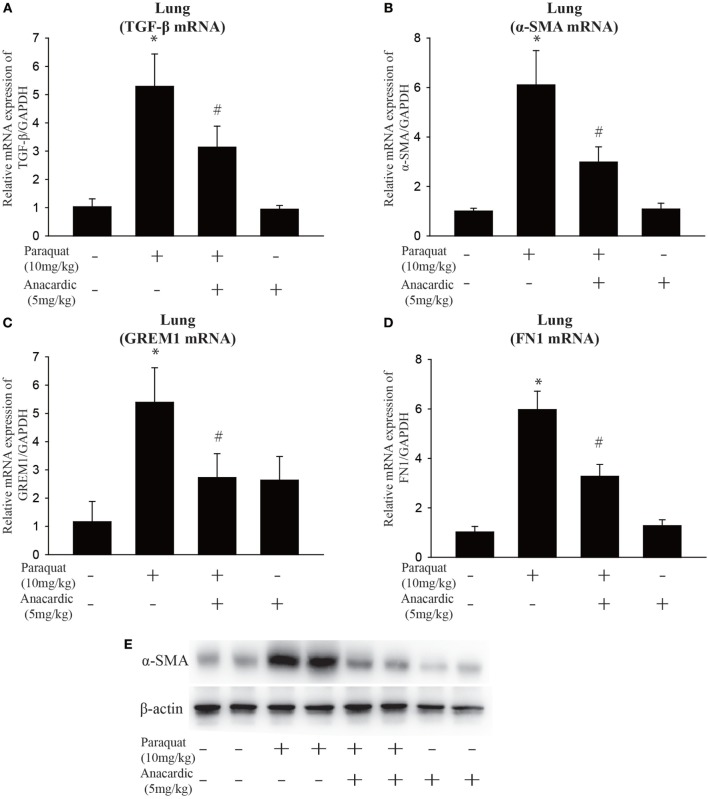
**Influence of histone acetyltransferase inhibition on gene expression of fibrotic markers**. Wild-type C57BL/6 male mice were treated with vehicle (saline) or 10 mg/kg of paraquat (PQ) for 1 month, and mice received anacardic acid (5 mg/kg) starting 1 h before PQ injection, 24 h and 15 days after PQ injection. **(A–D)** *TGF-*β, α-smooth muscle actin (α*-SMA*), *GREM1*, and *FN1* expression were determined by real-time PCR. **(E)** Western blot analysis of α*-SMA* expression in whole-lung lysates. Data are expressed as means ± SEM, *P < 0.05 versus vehicle and ^#^*P* < 0.05 versus PQ (*n* = 4–6).

Given the previous data showing *IL-6* play a vital role in pulmonary fibrosis, we next explore its regulation mechanism. HDAC inhibitors have been shown to influence interstitial fibrosis (47–49). To determine the effect of histone acetylation on *IL-6* expression, we measured *IL-6* mRNA and protein expression in the lung, serum, and macrophages. We observed that PQ-induced upregulation of *IL-6* mRNA and protein levels were further enhanced in the presence of the HDAC inhibitor VPA but reduced in the presence of the HAT inhibitor anacardic acid (Figures 4A–H). The ability to increase *IL-6* expression was also observed with other HDAC inhibitors in macrophages, as we can see that *IL-6* was significantly elevated in cells treated with the HDAC inhibitors suberoylanilide hydroxamic acid (SAHA) and scriptaid (Figure S4A in Supplementary Material). In addition, PQ-induced pulmonary acute inflammation was aggravated in the presence of VPA and mitigated in the presence of the anacardic acid (Figures 5A,B). For long-term (1 month) treatment, Masson’s trichrome and *COL1*α immunofluorescence in lung sections indicated that anacardic acid reversed PQ-induced pulmonary fibrosis (Figures 5C,D). The data are consistent to the observation that the mRNA and protein level of EMT-related genes *TGF-*β, α*-SMA, GREM1*, and *FN1* were decreased in the lung tissues from PQ-treated mice in the presence of anacardic acid (Figures 6A–E; Figure S4B in Supplementary Material). To determine the significance of HDACs in PQ-induced pulmonary fibrosis, we assessed the expression level of HDACs in the lung and macrophages. We found decreased the expression of *HDAC3* in PQ-treated lungs in a dose-dependent manner (Figure S5A in Supplementary Material). In macrophages treated with PQ, we also observed the loss of *HDAC3* expression in a dose-dependent and time-dependent manner (Figures S5B,C in Supplementary Material). While the mRNA level of HDACs 1, 2, 5, and 7 were significantly increased in the presence of PQ, and the expression of HDACs 4 and 8 remain unchanged (Figures S5D–I in Supplementary Material).

### Epigenetic Regulation of *IL-6* by Histone Acetylation

**Figure 7:**
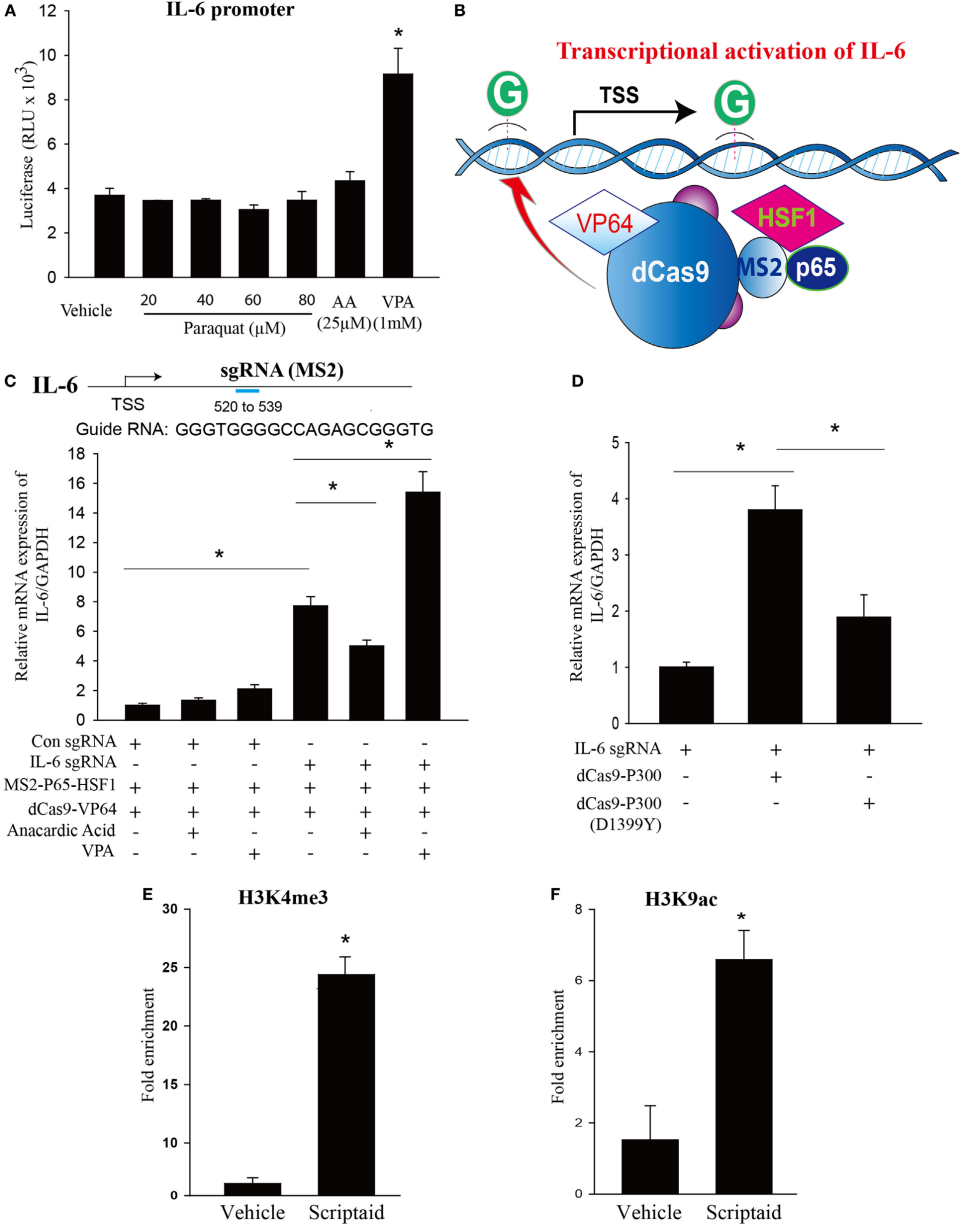
**Modulation of endogenous *IL-6* expression using CRISPR-ON, and the effects of histone deacetylase (HDAC) or histone acetyltransferase inhibition and epigenetic regulation of *IL-6* transcription by HDAC inhibition**. **(A)** HEK 293 T cells cotransfected with Firefly luciferase (LUC) reporter plasmids consisting of human *IL-6* promoter and *Renilla* luciferase as a transfection control were treated with vehicle (DMSO), paraquat (20, 40, 60, and 80 μM), anacardic acid (25 µM), or VPA (1mM) for 24 h, and luciferase activity determined using a Polarstar Omega (BMG Labtech, Durham, NC, USA) luminometer. **(B)** Schematic of the CRISPR-ON strategy to activate *IL-6* gene expression. **(C)** Guide RNA (gRNA) sequences and binding regions on the *IL-6* promoters. HEK-293 cells were transfected with either a control gRNA or gRNA targeting the proximal *IL-6* promoter regions in the presence or absence of VPA (1 mM) or anacardic acid (25 µM) for 24 h. **(D)** HEK-293 cells were co-transfected with IL-6 gRNA and dCas9-p300 or dCas9-p300 (D1399Y) plasmids. *IL-6* mRNA levels were determined by real-time PCR relative to GAPDH. **(E,F)** Macrophages treated with vehicle (DMSO) or scriptaid (3 µg/ml) were fixed with 1% formaldehyde for 10 min and chromatin prepared by enzymatic shearing for 10 min. ChIP was performed on isolated sheared chromatin using a negative control IgG and ChIP grade antibodies to H3K4 trimethylation (H3K4me3) or H3K9 acetylation (H3K9ac). Real-time PCR was performed on DNA purified from each of the ChIP reactions using primers specific for the *IL-6* promoter regions located upstream of *IL-6* transcriptional start site. HEK 293 T cells were plated in 12-well plates at a density of 0.5–1 × 10^6^ cells/well, and the data are representative of *n* > 5 from each group. Macrophages were plated in 100 mm dish at a density of 1–1.5 × 10^7^ cells/dish, and the data are representative of *n* > 5 from each group. Data are expressed as means ± SEM, *P < 0.05 (*n* = 4–6).

We next determined whether inhibition of HDACs or HATs affects *IL-6* promoter activity. We used *IL-6* promoter-luciferase reporter plasmids containing the proximal 5′ reporter region upstream of the transcription start site. The relative *IL-6* promoter was enhanced in the presence of HDAC inhibitor VPA, which remains unchanged in the presence of HAT inhibitor anacardic acid and PQ (Figure 7A). Given that the inability of PQ to modify *IL-6* promotor activity and the differences between promoter assay and native gene expression, we next assessed the epigenetic regulatory mechanisms of *IL-6* transcription.

As a tool to interrogate the epigenetic regulations of *IL-6* expression, we employed a CRISPR-ON strategy which based on the inactive Cas9 enzyme. As shown in Figures 7B,C, CRISPR-ON targeted to the *IL-6* promoter regions increased the expression of *IL-6*. The ability of CRISPR-ON to drive *IL-6* gene transcription was increased in the presence of VPA while attenuated in the presence of anacardic acid. P300 is a highly conserved acetyltransferase involved in a wide range of cellular processes. The fusion of p300 to dCas9 enables p300 to specifically target this enzyme to the endogenous promoters of *IL-6*. As shown in Figure 7D, the dCas9p300 Core fusion increased *IL-6* expression by transient cotransfection with sgRNA targeting the endogenous promoters of *IL-6* in human HEK293T cells. A single inactivating amino acid substitution within the HAT core domain of dCas9p300 Core [dCas9p300 Core (D1399Y)] abolished the transactivation capacity of the fusion protein, demonstrating that p300 HAT domain is sufficient to activate *IL-6* transcription.

Chromatin immunoprecipitation–qPCR experiments were employed to ascertain epigenetic changes at the *IL-6* promoter regions in macrophages. As shown in Figures 7E,F, marks of open chromatin structures including trimethylation of lysine 4 of histone H3 (H3K4me3) and acetylation of lysine 9 of histone H3 (H3K9ac) binding at the *IL-6* promoter region were significantly increased in the presence of HDAC inhibitor, scriptaid.

## Discussion

The purpose of this study was to explore the effect of *IL-6* on PQ-induced pulmonary fibrosis and further to interpret its underlying mechanisms. We found that PQ could dose-dependently and time-dependently increase *IL-6* mRNA expression from lung tissues and macrophages concomitant with the upregulation of protein expression. In addition to *IL-6* expression, PQ also harbored the ability to induce pulmonary acute inflammation and pulmonary fibrosis. Pulmonary fibrosis and fibrotic genes can be alleviated by blocked *IL-6* trans-signaling with recombinant soluble gp130Fc. *IL-6* release from macrophages in response to PQ mainly plays a central role in pulmonary fibrosis through enhanced EMT transition in pulmonary epithelial cells. Mechanistically, histone acetylation could enhance the transcription of *IL-6*, which is involved in the progression of PQ-induced pulmonary fibrosis. Histone acetylation didn’t alter the activity of transiently expressed *IL-6* promoter-luciferase constructs. ChIP–qPCR experiments revealed increased levels of H3K4me3 and H3K9ac at *IL-6* promoter sites in the presence of HDAC inhibitor, indicating that HDAC inhibitor could alter chromatin accessibility. As followed, the ability of CRISPR-ON to stimulate transcription of the endogenous *IL-6* genes was reduced in the presence of HAT inhibitor and enhanced in the presence of HDAC inhibitor. Targeting p300 HAT core domain to the endogenous promoters of *IL-6* significantly increased *IL-6* transcription. Together, our study uncovered the epigenetic regulatory mechanism of *IL-6* in macrophages that played a critical role in PQ-induced pulmonary fibrosis.

*Interleukin 6*, described as a cytokine regulating immune-neural-endocrine, participates in cell growth, differentiation, and function adjustment and plays an important role in the immune and inflammatory response. Although various cytokines are involved in the localized tissue injury or systemic damage, *IL-6* is a kind of important inflammatory mediator response to localized tissue injury. *IL-6*-deficient mice cannot mount a normal inflammatory response to localized tissue damage induced by turpentine treatment and the expression of induced acute phase proteins goes an apparent decline (50). *IL-6* binds IL-6R and associates with two molecules of gp130 to initiate the intracellular signaling cascade to exert its biological functions (51). A growing body of studies indicates that *IL-6* takes part in occurrence and development of fibrosis diseases (19, 20, 52, 53). Fibrosis is characterized by fibroblast aggregation, ECM deposition accompanied by disruption of the normal tissue structure and function caused by inflammation and injury (4, 54). Some cytokines involved in the formation of fibrosis diseases were confirmed by the experiments *in vitro* and *in vivo*. The role of cytokines in fibrosis formation has become a hot spot of research and *IL-6* has ability to promote fibrosis formation alone or together with *TNF-*α in bleomycin-induced pulmonary fibrosis. In addition, previous study indicates a possible association between sensitivity to bleomycin-induced fibrosis and inducibility of *IL-6* mRNA upon drug treatment (55).

*Interleukin-6* increased significantly in bleomycin-induced pulmonary fibrosis and other fibrosis diseases such as liver fibrosis, cystic fibrosis, and systemic scleroderma (56, 57). Therefore, *IL-6* is described not only as a classic proinflammatory cytokine but also as a profibrotic factor, even though the exact underlying mechanism remains a mystery (18, 23, 58). In our study, we demonstrated that *IL-6* overexpression in PQ-induced pulmonary fibrosis and blockade of GP130Fc reduced IL-6-induced fibrosis-related genes induction. Collectively, the above data suggested that *IL-6* did play an important role in pulmonary fibrosis, but the exact mechanism is elusive.

Despite a large amount of research investigating this area, it is rather surprising that it is still unknown at the molecular level how *IL-6* leads to fibrosis and which downstream signaling pathway contributes to this ECM deposition. Transdifferentiation of cardiac fibroblasts to the pathogenic myofibroblasts mediated by *IL-6* has been reported (54). In a rat model of cardiac hypertrophy by artery ligation, collagen synthesis and hypertrophy occur but inhibition of *IL-6* resulted in blockade of fibrosis and hypertrophy suggesting that *IL-6-*mediated collagen synthesis is driving the fibrosis and hypertrophy through a direct mechanism. However, in our study, we demonstrated that *IL-6* leads to fibrosis aggravation through an indirect mechanism that is mediated by EMT transition. Since *IL-6* plays a vital role in PQ-induced fibrosis, the mechanisms underlying the regulation of *IL-6* remain unknown. To this end, we next studied the epigenetic regulatory mechanism of *IL-6*.

Epigenetic modifications of chromatin are classic tools to regulate genes expression (24). Activity of HDAC family enzymes that catalyze the removal of diacetyl or acetyl groups from proteins was linked with development and progression of fibrosis (31, 59). While the functions of HAT are opposite. HDAC inhibitors, especially class I and II inhibitors, theoretically should increase histone acetylation and gene transcription. Our study shows that HDAC inhibitors promote the expression of *IL-6* mRNA and protein, thus PQ-induced pulmonary fibrosis was aggravated. In contrast, previous studies reported that the HDAC inhibitor mocetinostat decreased *IL-6* expression in congestive heart failure myocardium and cardiac fibroblasts (49, 60, 61). Increased HDAC activity has been found to be associated with fibrotic disorders, and the application of HDAC inhibitors could provide a potential benefit in the treatment of fibrotic disorders (62). In human lung fibroblasts, HDAC inhibitor TSA reduced *TGF-*β-induced α*-SMA* and *collagen 1-*α expression (63). While other investigators have found the reduced HDAC activity and increased HAT activity in inflammatory lung disease, especially *HDAC2* expression and activity was reduced in COPD (27, 64). The treatment of HDAC inhibitor TSA caused right ventricle dysfunction and fibrosis in a rat model of pulmonary artery binding (65). Increased oxidative stress could suppress HDAC activity and expression, which is important to reduce inflammatory gene expression in the lung and macrophages, thus HDAC activator might have important clinical implications in severe asthma and cystic fibrosis (25). The reasons underlying this discrepancy are not known but may be due to a model-dependent effect or a cell type-dependent effect or HDAC inhibitor type-dependent effect. We found that multiple HDAC inhibitors effectively increase *IL-6* expression in macrophages *in vitro* and in PQ-treated lung tissues *in vivo*. In addition, the ability of HDAC inhibitors to modify gene expression is complex, previous studies demonstrated that approximately 30% of the transcriptome are regulated by HDACs with equal proportion of the upregulation and downregulation in gene expression, and the pattern and direction of changes in gene expression are different and depended on cell types (33, 34). This is partially due to the fact that HDAC inhibitors can significantly increase the deacetylation of histones at multiple genomic DNA regions (33). VPA inhibits HDAC classes I (*HDAC1, 2, 3*, and *8*) and II, while scriptaid and SAHA specifically inhibit HDAC class I. We found low expression of *HDAC3, 4*, and *8* and overexpression of *HDAC1, 2, 5*, and *7* in PQ-treated macrophages and lungs. Whether the increased IL-6 expression is connected with these decreased HDACs is not fully understood. On the contrary, HAT inhibitor anacardic acid inhibited the expression of *IL-6* and alleviated fibrosis.

To interrogate the relationship between the HDAC and HAT with *IL-6* regulation, here we have employed CRISPR-ON which on the basis of CRISPR/Cas9 gene regulation system. The efficiency of CRISPR-ON could be influenced by chromatin remodeling with the help of RNA guides and dCAS9. We found that the HAT inhibitor anacardic acid reduced the ability of CRISPR-ON to increase *IL-6* mRNA expression and HDAC inhibitor VPA increased the ability of CRISPR-ON to increase *IL-6* mRNA expression. These results indicate that the chromatin remodeling controlled by histone modifications around the promoter sequences of *IL-6* gene is important in regulating its transcriptional activity. As a potent easily programmable tool to regulate acetylation at targeted endogenous loci, dCas9p300 Core fusion protein leading to manipulate targeted genes with the help of intrinsic p300 Core acetyltransferase activity. In our study, dCas9p300 Core fusion protein increased *IL-6* mRNA expression and the dCas9p300 Core (D1399Y) acetyltransferase-null mutant had lower ability to increase the *IL-6* mRNA expression. Thus, transcriptional coactivator p300/CBP, with HAT activity, plays a role in influencing the *IL-6* expression (66). These results are also supported by ChIP analysis, which revealed that HDAC inhibitors scriptaid increased the presence of histones bearing activation marks (H3K4me3 and H3K9ac) at the *IL-6* promoter regions, suggesting that chromatin accessibility in the region of the *IL-6* promoter is increased by HDAC inhibition.

In summary, our study reveals the role of *IL-6* in PQ-induced pulmonary fibrosis and a new epigenetic mechanism that controls the transcription of *IL-6 in macrophages*. We have found that the expression of *IL-6* mRNA and protein were potently increased in lungs and macrophages treated with PQ. Blockade of *IL-6* trans-signaling by GP130Fc attenuated PQ-induced pulmonary fibrosis. Acetylation of histones through the increased binding of HAT p300 to the promoter regions of *IL-6* elevates gene transcription. HAT inhibitor reduced *IL-6* transcription. In addition, HAT inhibitor reduced the expression of fibrosis-related genes and mitigated the degree of PQ-induced pulmonary fibrosis. Therefore, histone acetylation potently regulates the expression of *IL-6* gene in macrophages which may be of significance in the treatment of diseases that result from the overproduction of *IL-6*, such as PQ-induced pulmonary fibrosis.

## Author Contributions

FC and DF designed the study. Lingli H, YY, HH, HF, LH, CY, and KL performed the laboratory experiments. Lingli H performed the statistical analysis and wrote the draft. FC made significant conceptual contributions to the manuscript and reviewed the final version of the paper. All the authors provided intellectual content and approved the final version of the paper.

## Conflict of Interest Statement

The authors declare that the research was conducted in the absence of any commercial or financial relationships that could be construed as a potential conflict of interest.

## Supplementary Material

Figure S1 **Expression of inflammation-related genes in lung from paraquat (PQ)-treated mice**. Wild-type C57BL/6 male mice were treated with vehicle (saline), 10 or 50 mg/kg of PQ for 3 days. **(A)** *IL-1*β, **(B)** *IL-8*, **(C)** *COX-2*, **(D)** *MMP9*, **(E)** *TNF-*α, and **(F)** *VCAM1* mRNA expression in the lung were measured by real-time PCR. Data are expressed as mean ± SEM, **P* < 0.05 versus vehicle (*n* = 3–6).

Figure S2 **Expression of *interleukin 6 (IL-6)* mRNA in paraquat (PQ)-treated 16HBE and HLF**. **(A)** 16HBE and **(B)** HLF were treated with increasing concentrations of PQ for 24 h, and *IL-6* mRNA expression was determined by real-time PCR. 16HBE and HLF cells were plated in six-well plates at a density of 1.5–2 × 10^6^ cells/well, and the data are representative of *n* > 5 from each group. Data are expressed as mean ± SEM (*n* = 3–6).

Figure S3 **Influence of recombinant *interleukin 6 (IL-6)* had no impact on the expression of fibrotic genes in fibroblasts**. Fibroblasts 3T3 were treated with vehicle or recombinant IL-6 10 ng/ml for 48 h. And **(A)** α-smooth muscle actin (α*-SMA*), **(B)** *GREM1*, and **(C)** *FN1* expression were determined by real-time PCR. Fibroblasts 3T3 were plated in six-well plates at a density of 1.5–2 × 10^6^ cells/well, and the data are representative of *n* > 5 from each group. Data are expressed as mean ± SEM (*n* = 3–6).

Figure S4 **Regulation of *interleukin 6 (IL-6)* expression by histone deacetylase inhibition and the densitometry analysis of α-SMA in PQ and anacardic acid treated mice lung**. **(A)** Macrophages were treated with vehicle or scriptaid 3 mg/ml, suberoylanilide hydroxamic acid 10 mM for 24 h. *IL-6* mRNA was determined by real-time PCR. Data are expressed as mean ± SEM, **P* < 0.001 versus vehicle (*n* = 3–6). **(B)** Densitometry analysis of α-smooth muscle actin (α*-SMA*) versus β-actin in lungs from control or paraquat (PQ) or anacardic acid (5 mg/kg) treated mice lung. Results are representative of at least three to five separate experiments. Data are expressed as means ± SEM, **P* < 0.05 versus control, and^#^ *P* < 0.05 versus PQ.

Figure S5 **Expression of histone deacetylase (HDAC) mRNA in paraquat (PQ)-treated lung and macrophages**. **(A)** *HDAC3* mRNA expression was measured in lung from control and day 3 PQ-treated mice. **(B–I)** The relative expression of *HDAC1, HDAC2, HDAC4, HDAC5, HDAC7*, and *HDAC8* were determined by real-time PCR relative to GAPDH in PQ-treated macrophages. Data are expressed as mean ± SEM, **P* < 0.05 versus vehicle (*n* = 4–6).
