# Supplementary figures and images for: Epigenetic Regulation of Interleukin 6 by Histone Acetylation in Macrophages and Its Role in Paraquat-Induced Pulmonary Fibrosis
Source: Front Immunol. 2017 Jan 30;7:696. doi: 10.3389/fimmu.2016.00696 (PMC5276821; doi:10.3389/fimmu.2016.00696)

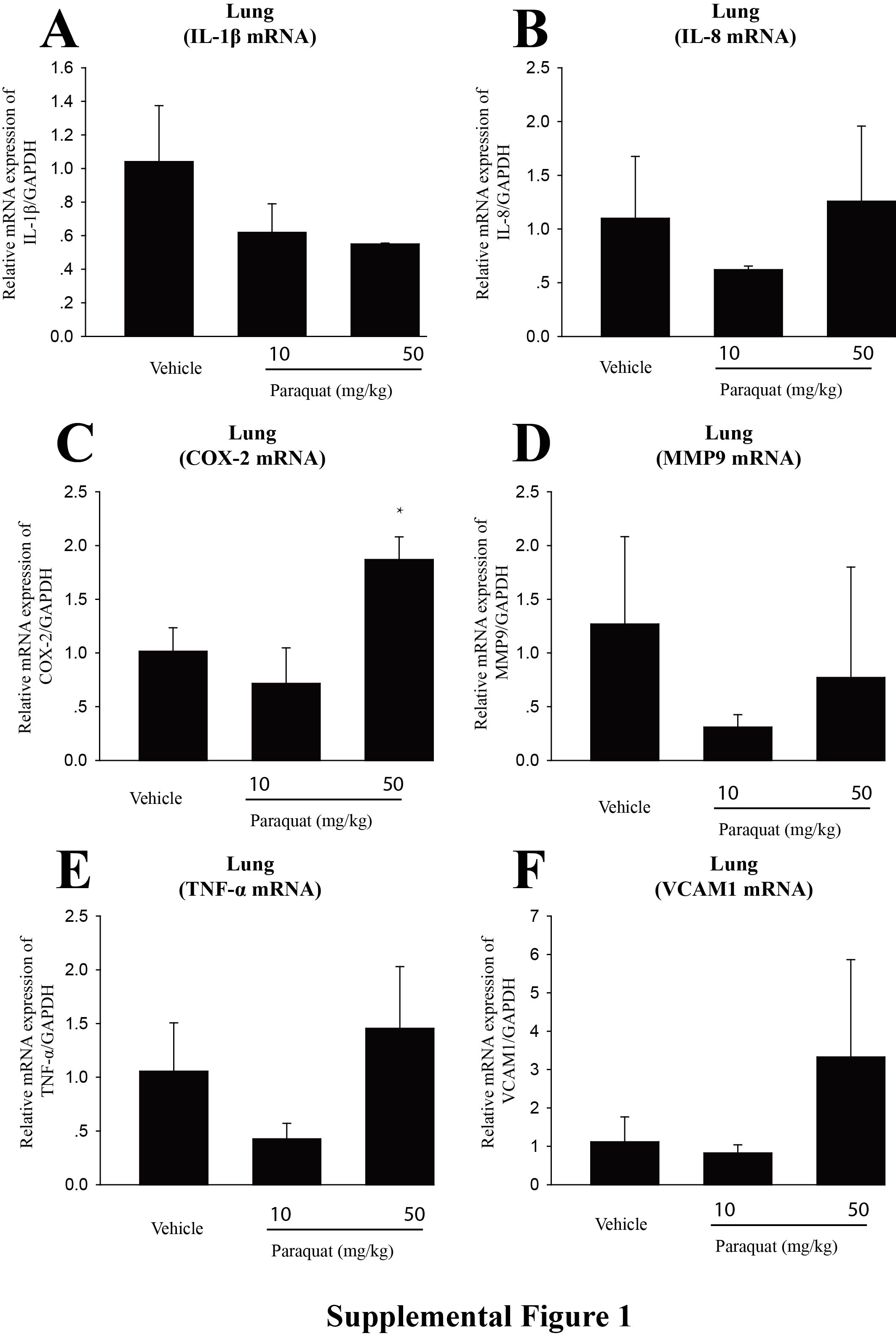

Supplement: Figure S1 — Expression of inflammation-related genes in lung from paraquat (PQ)-treated mice. Wild-type C57BL/6 male mice were treated with vehicle (saline), 10 or 50 mg/kg of PQ for 3 days. (A) IL-1β, (B) IL-8, (C) COX-2, (D) MMP9, (E) TNF-α, and (F) VCAM1 mRNA expression in the lung were measured by real-time PCR. Data are expressed as mean ± SEM, *P < 0.05 versus vehicle (n = 3–6). [file Image_1.JPEG]

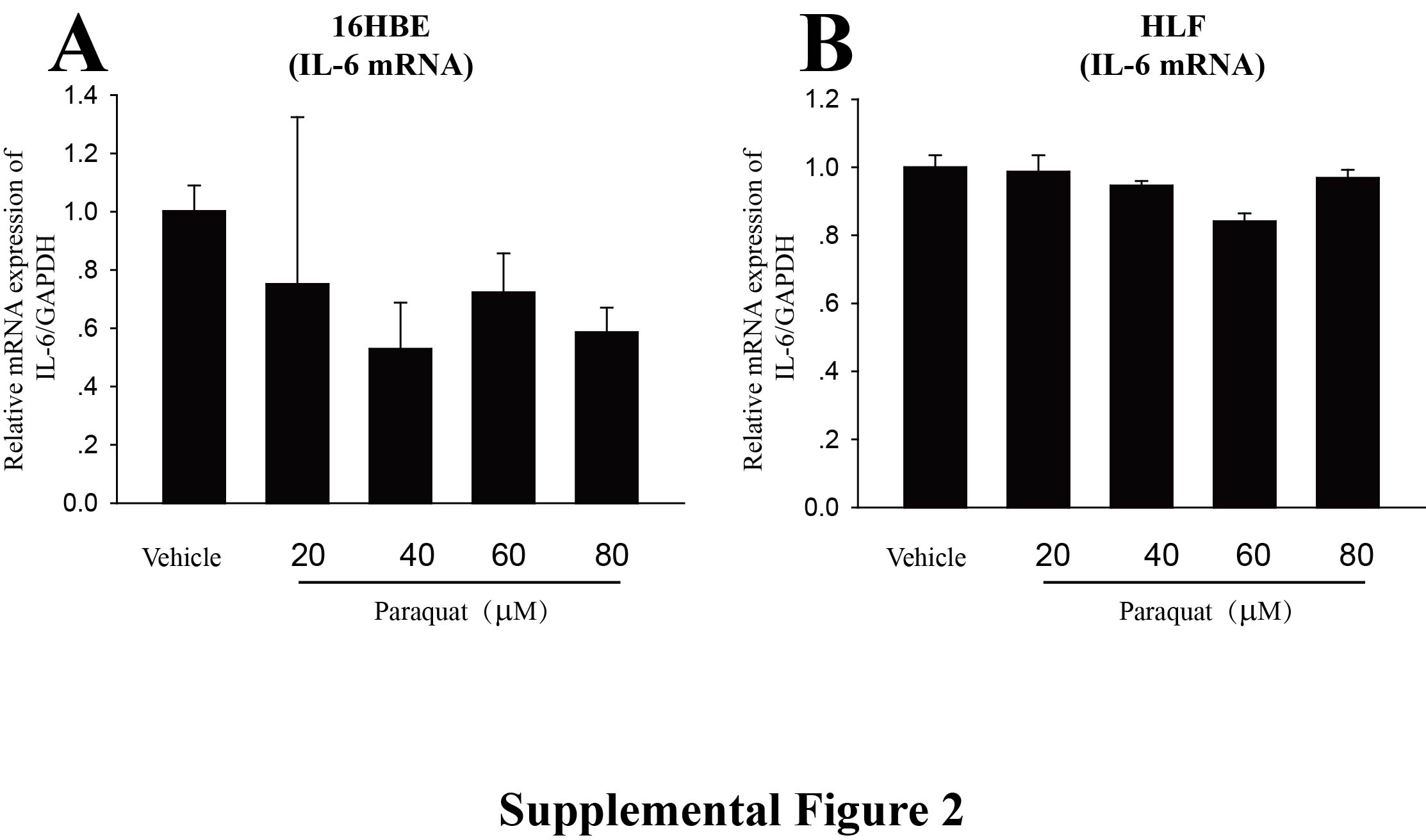

Supplement: Figure S2 — Expression of interleukin 6 (IL-6) mRNA in paraquat (PQ)-treated 16HBE and HLF. (A) 16HBE and (B) HLF were treated with increasing concentrations of PQ for 24 h, and IL-6 mRNA expression was determined by real-time PCR. 16HBE and HLF cells were plated in six-well plates at a density of 1.5–2 × 10^6^ cells/well, and the data are representative of n > 5 from each group. Data are expressed as mean ± SEM (n = 3–6). [file Image_2.JPEG]

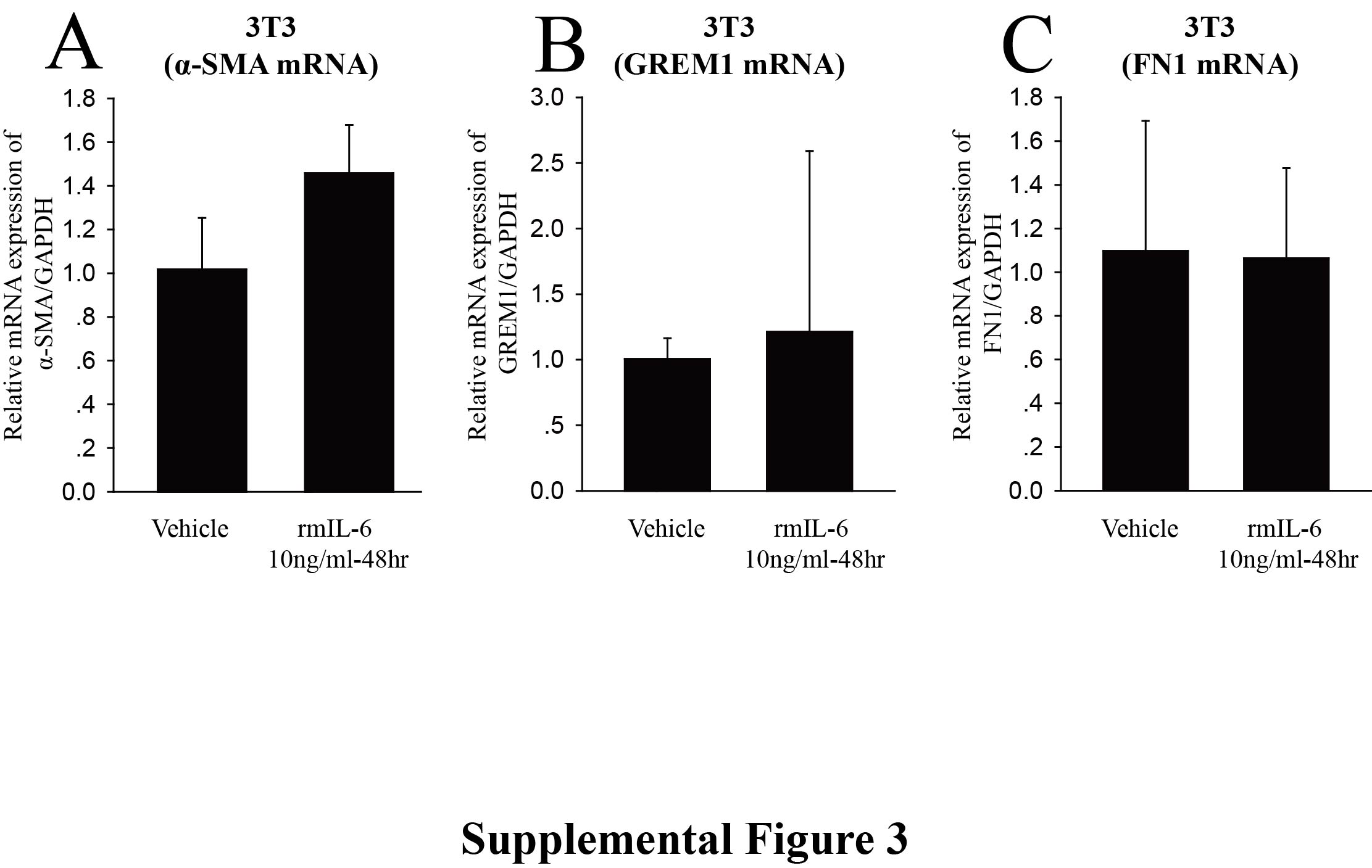

Supplement: Figure S3 — Influence of recombinant interleukin 6 (IL-6) had no impact on the expression of fibrotic genes in fibroblasts. Fibroblasts 3T3 were treated with vehicle or recombinant IL-6 10 ng/ml for 48 h. And (A) α-smooth muscle actin (α-SMA), (B) GREM1, and (C) FN1 expression were determined by real-time PCR. Fibroblasts 3T3 were plated in six-well plates at a density of 1.5–2 × 10^6^ cells/well, and the data are representative of n > 5 from each group. Data are expressed as mean ± SEM (n = 3–6). [file Image_3.JPEG]

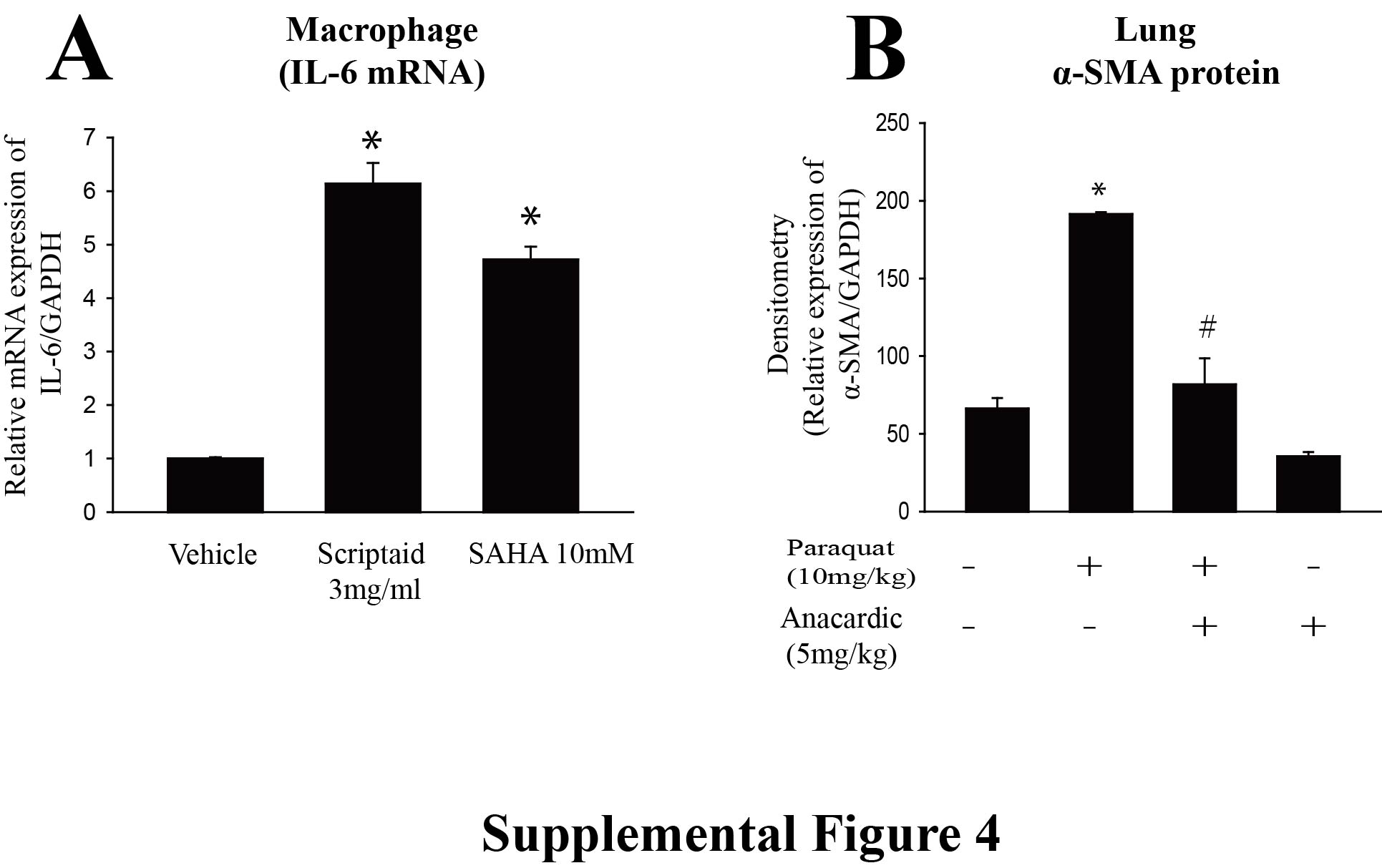

Supplement: Figure S4 — Regulation of interleukin 6 (IL-6) expression by histone deacetylase inhibition and the densitometry analysis of α-SMA in PQ and anacardic acid treated mice lung. (A) Macrophages were treated with vehicle or scriptaid 3 mg/ml, suberoylanilide hydroxamic acid 10 mM for 24 h. IL-6 mRNA was determined by real-time PCR. Data are expressed as mean ± SEM, *P < 0.001 versus vehicle (n = 3–6). (B) Densitometry analysis of α-smooth muscle actin (α-SMA) versus β-actin in lungs from control or paraquat (PQ) or anacardic acid (5 mg/kg) treated mice lung. Results are representative of at least three to five separate experiments. Data are expressed as means ± SEM, *P < 0.05 versus control, and# P < 0.05 versus PQ. [file Image_4.JPEG]

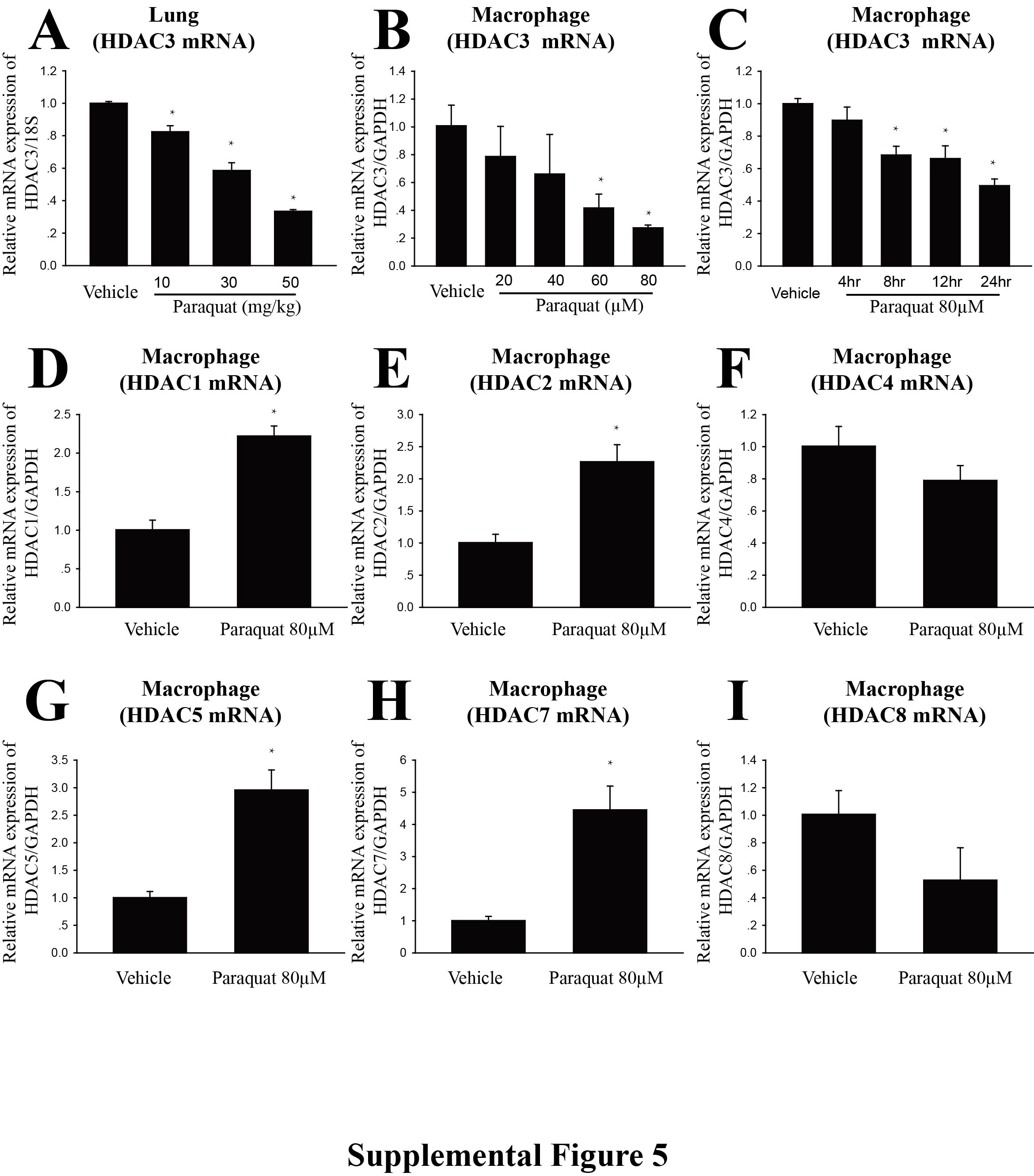

Supplement: Figure S5 — Expression of histone deacetylase (HDAC) mRNA in paraquat (PQ)-treated lung and macrophages. (A) HDAC3 mRNA expression was measured in lung from control and day 3 PQ-treated mice. (B–I) The relative expression of HDAC1, HDAC2, HDAC4, HDAC5, HDAC7, and HDAC8 were determined by real-time PCR relative to GAPDH in PQ-treated macrophages. Data are expressed as mean ± SEM, *P < 0.05 versus vehicle (n = 4–6). [file Image_5.JPEG]
